# Ecological differentiation at the polar frontier: metabolic strategies of endemic and cosmopolitan bacteria in Antarctic microbial mats

**DOI:** 10.3389/fmicb.2026.1835163

**Published:** 2026-06-24

**Authors:** Dorota Górniak, Aleksander Świątecki, Jakub Kowalik, Jakub Grzesiak, Justyna Możejko-Ciesielska, Marek K. Zdanowski

**Affiliations:** 1 Faculty of Biology and Biotechnology, University of Warmia and Mazury in Olsztyn, Olsztyn, Poland; 2 Department of Animal Taxonomy and Ecology, Adam Mickiewicz University, Poznań, Poland; 3 Institute of Biochemistry and Biophysics, Polish Academy of Sciences, Warszawa, Poland

**Keywords:** Antarctica, biogeography, endemic and cosmopolitan bacteria, environmental tolerance, metabolic activity, microbial mats

## Abstract

This study is the first to characterize the metabolic strategies of endemic and cosmopolitan bacterial species in Antarctic microbial mats. Of the 43 isolates obtained, 21 strains were identified as belonging to 12 endemic (EN) bacterial species, while 22 strains represented 16 cosmopolitan (CO) species. Metabolic profiles for these strains were determined using the Biolog Gen III microarray metabolic fingerprinting test. The results suggest a possible relationship between patterns of metabolic activity, environmental tolerance, and the biogeographic range of the species. CO strains showed statistically significantly higher metabolic activity, utilizing approximately half of the substrates, whereas EN strains utilized one-third. Statistically significant differences were observed in the utilization of sugar alcohols, amino sugars, phosphosugars, amino acids, and sugar acids. Among CO species, strains with exceptionally broad metabolic potential and high resistance to environmental factors were identified. The results confirmed the hypothesis that the metabolic strategies of bacteria in microbial mats may be determined by the original biogeographic range of the species. As indicated by this study, the presence of cosmopolitan species in polar environments alter the structure of native microbial communities. Their migration to isolated environments may contributes to the biotic homogenization of microbiomes globally, potentially leading to the gradual displacement of endemic species. Consequently, this phenomenon may influence key ecological processes in polar ecosystems, including matter cycling and the functioning of microbial communities. The current study contributes to the discussion on contemporary microbial biogeography, indicating that the geographic ranges of species may be related to metabolic strategies and environmental adaptations.

## Graphical Abstract

**Figure:**
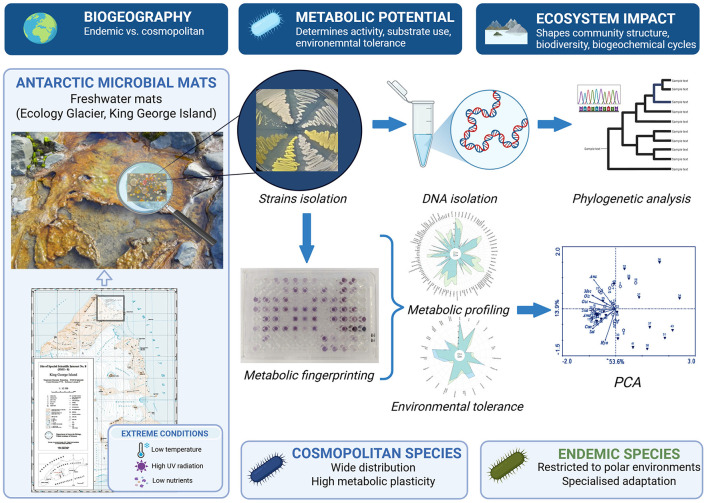
Created in BioRender. Możejko-Ciesielska, J. (2026) https://BioRender.com/fz0sr3c.

## 1 Introduction

Antarctica is the most isolated region of the Earth's cryosphere. The limited availability of liquid water in terrestrial Antarctica is a crucial factor determining the development of microbial communities (Hawes et al., 2023). Polar environments, i.e., areas located at high latitudes are characterized by extreme conditions, including low temperatures, high UV radiation, poor nutrient availability, and strong pressure from cyclical freezing and thawing (Králová et al., 2021). The selective nature of these factors promotes the survival and development of only those microorganisms with high adaptive potential (Králová et al., 2021). Microbial mats are the dominant component of the total biomass of terrestrial polar environments (Quesada et al., 2009; Jungblut et al., 2016; Velázquez et al., 2016). They are characterized by a complex community structure comprising autotrophic (photo- and chemo-lithotrophic), heterotrophic, and photoheterotrophic microorganisms (Van Trappen et al., 2002; Prieto-Barajas et al., 2018). A distinguishing feature of these ecosystems is their self-sufficiency and autonomy associated with the complementarity of primary and secondary production and the regeneration of nutrients. The functional foundation of mat microbial communities is the stable, integrated metabolic activity of the entire microbial community. Internal metabolic balance is of key importance for microbial mat homeostasis at various functional levels (Dillon et al., 2020; Lezcano et al., 2022). Research indicates that heterotrophic bacterial communities play a fundamental role in nutrient recycling in polar microbial mats, which is crucial for the functioning of the entire community (Valdespino-Castillo et al., 2018). Due to the limited availability of nutrient substrates, the metabolism of microorganisms of polar mats is adapted to these trophic conditions. These microorganisms typically have a narrow metabolic range and primarily utilize simple, readily utilized substrates (Valdespino-Castillo et al., 2018). With increased geographic ranges, cosmopolitan heterotrophs reach these oligotrophic environments and actively participate in the recycling of organic matter, thereby influencing mat structure (Prieto-Barajas et al., 2018). As a result, both local species and taxa with global ranges may be present in polar microbial mats (Tytgat et al., 2023).

Research on the biogeography of microorganisms and their presence in various environments focuses on species endemism and cosmopolitanism, as well as on the biotic homogenisation of microbiomes on a global scale (Martiny et al., 2006; James et al., 2024). Baas Becking's classic concept (1934) of “everything is everywhere, but the environment selects” underpins considerations of biodiversity and the formation of microbial consortia. This approach highlights both the ubiquity of microbial cosmopolitanism and the importance of environmental factors in the selection of prokaryotic strains. The opposing concept, however, points to the endemic nature of microorganisms in isolated environments (Boenigk et al., 2006; Bienhold et al., 2016). In the microbial communities of circumpolar environments, attention is given to the unique characteristics of polar microorganisms, which originate from permanently or periodically frozen ecosystems (Hahn et al., 2015). In an endemic model, the isolation of these ecosystems promotes the development of characteristic ecotaxonomic patterns of native microorganisms, which have a limited biogeographical range (Nemergut et al., 2011; Fontaneto and Hortal, 2012; Tytgat et al., 2023). Adaptation to extreme conditions is emphasized as the primary mechanism shaping the structure and functioning of polar microbial communities (Gawor et al., 2016; Zhang et al., 2021, 2025). Both endemism and cosmopolitanism of polar prokaryotes are considered typical characteristics of heterogeneous microbial consortia (Van Horn et al., 2016; Varliero et al., 2024). Progressive global climate change is weakening and shifting biogeographical boundaries, promoting the colonization of previously isolated extreme environments by cosmopolitan prokaryotes (Bargagli and Rota, 2024; Rizzo et al., 2024; Zucconi et al., 2025; Mao et al., 2026). As demonstrated by (Pessi et al. 2018), microorganisms with a cosmopolitan range constitute a significant proportion of Antarctic microbial mats. Genome plasticity plays a crucial role in species adaptation to extreme environments. The overlap between the biogeographical ranges of endemic and cosmopolitan species increases the gene pool of microbial consortia (James et al., 2024). Research on polar environment microbial communities insufficiently considers biogeography and the distribution of cosmopolitan and endemic bacterial species, which is particularly important in the context of progressive global climate change and the increased susceptibility of previously isolated environments to colonization. The spread of alien species in polar environments disrupts the functioning of stable microbial communities and adversely affects endemic prokaryotic populations (Cowan et al., 2011; Kleinteich et al., 2017; Leihy et al., 2023; Zucconi et al., 2025). These processes may lead to changes in the structure of the polar microbiome, such as declines in endemic population sizes and alterations in the genomic, metabolic, and physiological characteristics of microorganisms (Kleinteich et al., 2017; Muñoz-Hisado et al., 2025; Swiatecki et al., 2026).

This study presents the results of research on Antarctic strains isolated from freshwater polar microbial mats found in ephemeral reservoirs of the proglacial zone of the Ecology Glacier on King George Island in the marine Antarctic. Microarray metabolic fingerprinting was used to examine the metabolic activity of the strains and their tolerance to environmental factors. It was hypothesized that taxonomic structure, shaped by different geographical ranges, is reflected in the diversity of metabolic pathways and adaptations to extreme environments.

## 2 Materials and methods

### 2.1 Bacterial strains and molecular identification

**Figure 1:**
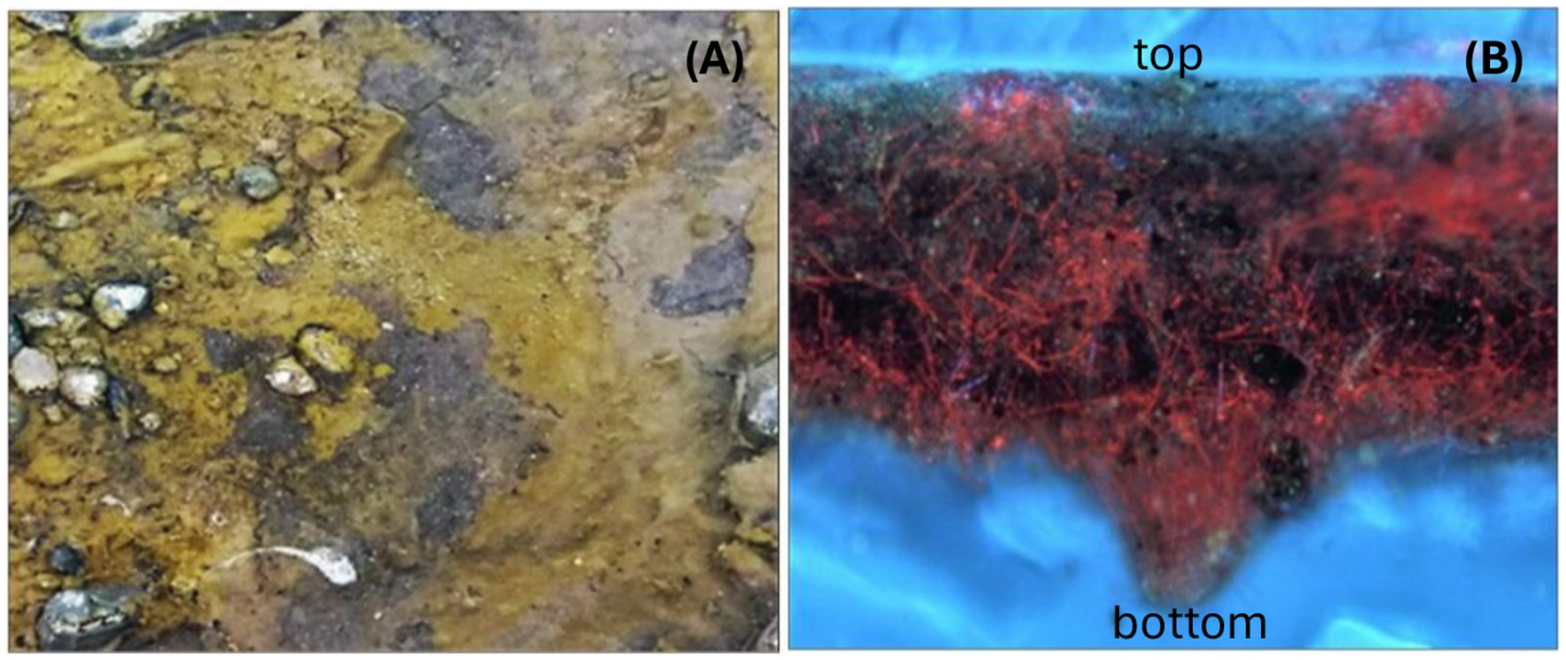
Macroscopic **(A)** and microscopic **(B)** (cross-section) structure of microbial mats (an example).

The study examined 43 bacterial strains derived from microbial mats developing in freshwater reservoirs of the periglacial zone of the Ecology Glacier. Figure 1 presents examples of the macroscopic and microscopic structures (cross-sections) of the microbial mats studied. After sampling, bacterial cultures were incubated at 7 °C for 1 month and inspected every third day for colony development and growth. The optimal growth temperature of all cultures was determined based on optimization tests. The optimal temperature for strain cultivation was determined experimentally by analyzing growth kinetics across a temperature gradient. Growth rates were measured nephelometrically at six points (5 °C−30 °C, in 5 °C increments) at 12-h intervals over 7 days. For the tested bacteria, the optimal temperature was 20 °C. The collection of microbial mat samples, as well as the isolation and identification of bacterial strains, are described in detail in a paper by (Górniak et al. 2025). For taxonomic identification of the strains, 16S rRNA gene sequencing was performed using the primers 27f 5′-GAG TTT GAT CCT GGC TCA G3′) and rp2 5′-ACG GCT ACC TTG TTA CGA CTT3′) in a PCR reaction. These primers amplify a fragment of about 1.5 kb in length corresponding to the nearly full-length bacterial 16S rRNA gene. Nucleotide sequences were compared by BLAST with those in the NCBI database (Johnson et al., 2008) and with the RDP classifier online programme (Cole et al., 2009).

### 2.2 Microarray metabolic fingerprinting

The BIOLOG Gen III test (Biolog Inc., Hayward, California, USA) is a commercial microbiological system used to assess the metabolic potential of bacterial strains. The method is based on analyzing microorganisms' ability to utilize various carbon sources and their responses to a set of chemical compounds, including inhibitors and salts. Despite the broad scope of the biochemical and functional analyses, the BIOLOG Gen III test does not account for physical environmental factors such as cyclic freezing and thawing or UV radiation. Although BIOLOG assays are conducted under highly standardized laboratory conditions, they capture a range of physiological traits that may potentially be expressed *in situ*.

Bacterial metabolism in the wells of the microtest plate produces a colored product, with the color intensity indicating the strain's metabolic activity. The test involves ninety-four different substrates and reactions, including the utilization of carbohydrates, amino acids, and carboxylic acids; respiration under conditions of variable pH and salinity; and the presence of various growth inhibitors. On this basis, it is possible to determine the metabolic and physiological profiles of the tested strain. The tests were performed in accordance with the manufacturer's recommendations. Bacterial suspensions were prepared in GEN III MicroPlate IF C inoculation fluid until 90% transmittance was achieved. Each microplate well was filled with 100 μl of the suspension. The plates were incubated in the dark at 20 °C, and the color intensity was measured at 590 nm using an OmniLog microplate reader, with each measurement performed in five replicates using the same inoculum.

### 2.3 Statistics

All data were statistically analyzed using the Statistica v. 13.3 programme (StatSoft Inc., Tulsa, OK, United States). Differences between groups were assessed using one-way analysis of variance (ANOVA), while for data that did not meet the assumptions of normal distribution, the non-parametric Kruskal–Wallis test was used. Correlations were examined using linear and non-linear regression analyses. Canoco for Windows v. 4.5 software (ter Braak and Šmilauer, 2002) was used for principal component analysis (PCA). The results were visualized as radar charts in the R environment, using the ggplot2 package.

## 3 Results

### 3.1 Taxonomic composition

**Figure 2:**
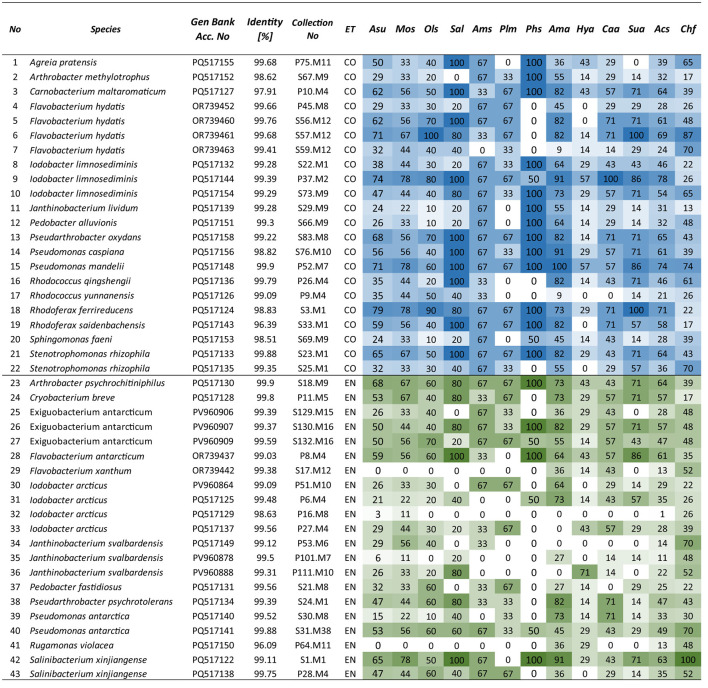
Metabolic activity of bacterial strains and their tolerance to chemical factors (% positive reactions). ET, ecological type of species; CO, cosmopolitan strains; EN, endemic strains; Asu, all sugars; Mos, monosugars; Ols, oligosugars; Sal, sugar alcohols; Ams, aminosugars; Plm, polymers; Phs, phosphosugars; Ama, amino acids; Hya, hydroxylic acids; Caa, carboxylic acids; Sua, sugar acids; Acs, all carbon sources; Chf, chemical factors.

Molecularly identified bacterial strains were assigned to two groups based on their documented ecogeographic distribution, using current data from public phylogenetic databases (Silva, RDP, NCBI GenBank). However, this division has certain limitations resulting from the current state of knowledge. Species with records currently limited to permanently cold, cryosphere-associated, or polar environments were classified as endemic. The classification used by the authors is not based on the classic, strict definition of biogeographic endemism, but reflects the current state of knowledge regarding the geographic distribution of individual taxa. These ranges were established solely based on records deposited in global databases and a critical review of the available literature. Species with a widespread distribution, common in various environments, were defined as cosmopolitan. This division is also limited by the uneven degree of exploration of different ecological niches worldwide. As some of the tested bacterial strains were not clearly identified to the species level, caution is required in the interpretation and extrapolation of the results. The observed physiological characteristics could be considered both in the context of the unique properties of specific isolates and as representative of the identified ecotypes. The adopted classification scheme assigned 21 of the 43 strains to 12 endemic species (EN), while the remaining 22 strains were identified as 16 cosmopolitan species (CO; Figure 2).

### 3.2 Metabolic profiling

**Figure 3:**
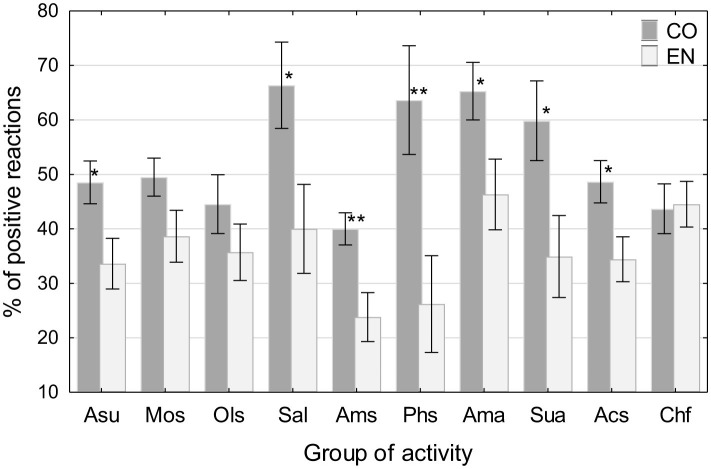
Metabolic activity and tolerance to chemical factors of cosmopolitan (CO) and endemic (EN) bacterial strains. Asu, all sugars; Mos, monosugars; Ols, oligosugars; Sal, sugar alcohols; Ams, aminosugars; Plm, polymers; Phs, phosphosugars; Ama, amino acids; Hya, hydroxylic acids; Caa, carboxylic acids; Sua, sugar acids; Acs, all carbon sources; Chf, chemical factors; *p < 0.05; **p < 0.01.

**Figure 4:**
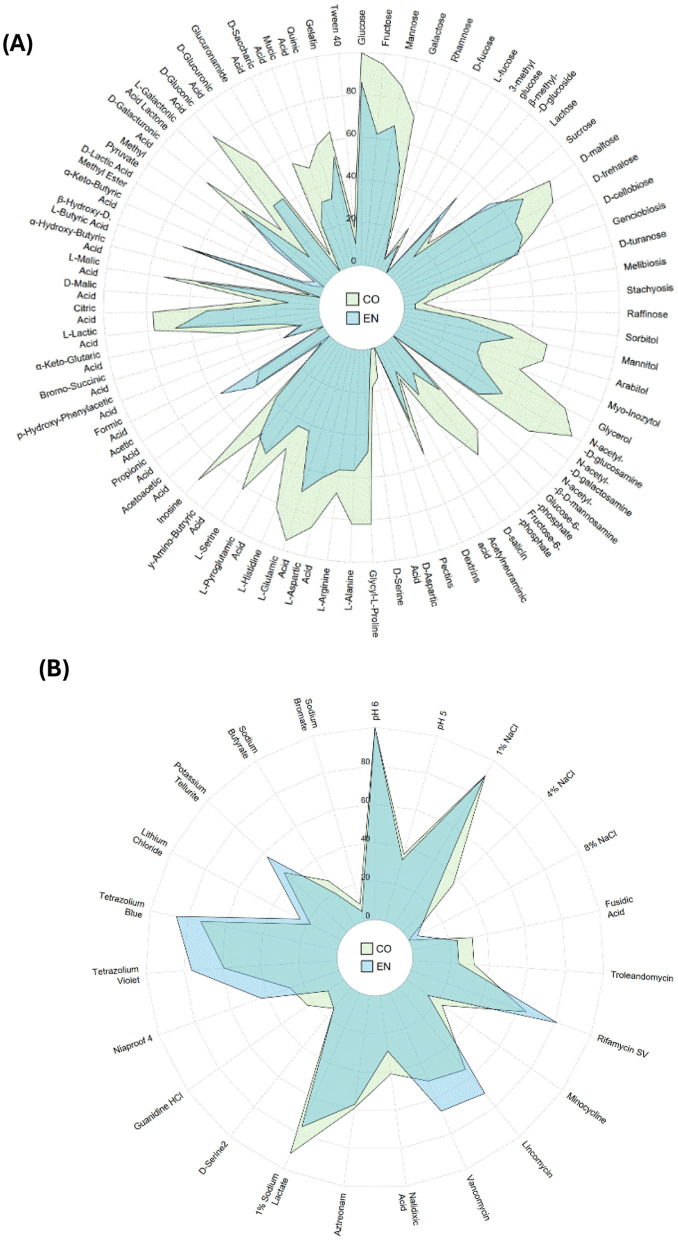
Metabolic activity **(A)** and tolerance to chemical factors **(B)** of CO and EN bacterial strains (percentage of active strains).

The activity in utilizing various carbon sources among bacterial strains derived from microbial mats was high, with an average of over 40% of substrates being metabolized (Figure 2). Exhibiting a similar metabolic pattern, CO strains showed higher, statistically significant (*p* < 0.05) activity, with an average of 49% of substrates, whereas EN strains metabolized an average of 35% of total substrates. Similar patterns, namely higher activity of CO strains than EN strains, were observed across the isolated carbon sources. Statistically significant differences were noted in the utilization of sugar alcohols (*p* < 0.05), amino sugars (*p* < 0.01), phosphosugars (*p* < 0.01), amino acids (*p* < 0.05), and sugar acids (*p* < 0.05). With regard to monosaccharides and oligosaccharides, no significant difference was observed in their assimilation by strains from the isolated ecotypes (Figure 3). Of the nine monosaccharides, glucose, fructose, mannose and galactose were the most readily utilized, while fucose and rhamnose were catabolized less frequently. However, none of the strains metabolized 3-methyl glucose. EN strains utilized ß-methyl-D-glucoside as a carbon source more readily than CO strains. Of the 10 oligosaccharides, the strains derived from microbial mats most readily utilized five, namely D-maltose, D-trehalose, D-cellobiose, gentiobiose, and sucrose (Figure 4A). D-turanose, lactose, melibiose, and raffinose were utilized less frequently. Sporadically, bacteria utilized stachyose, which was metabolized by only 7% of the strains. A statistically significant difference between the CO and EN ecotypes under study was observed in sugar alcohol metabolism. These substrates were utilized more readily by CO strains as carbon sources. Among the five sugar alcohols, these bacteria most readily utilized glycerol (86% of strains), whereas only 43% of EN strains exhibited this activity. Similar proportions were noted for the utilization of arabitol, sorbitol, mannitol, and myo-inositol (Figure 4A). A statistically significant (*p* < 0.01) difference in the metabolism of amino sugars by the isolated bacterial groups was demonstrated (Figure 3). Two of the three amino sugars used in the BIOLOG Gen III test (N-acetyl-D-glucosamine and N-acetyl-D-galactosamine) were utilized by the majority (87%) of cosmopolitan bacterial strains and 50% of strains classified as endemic bacteria. Notably, bacteria from microbial mats did not utilize N-acetyl-β-D-mannosamine as a substrate. A statistically significant difference (*p* < 0.01) in the metabolism of phosphosugars by microbial mat bacteria was observed, with CO strains decomposing them more frequently (Figure 3). Over two-thirds of these strains used phosphosugars as potential carbon and phosphorus sources, whereas only one-third of EN strains metabolized these substrates. Of the 11 amino acids tested, four compounds were used most frequently: L-glutamic acid, L-aspartic acid, L-serine, and L-alanine, which were metabolized by over 70% of all strains. Notably, the number of strains (less than 20%) that metabolize D-aspartic acid and D-serine was small. A higher, statistically significant (*p* < 0.05) metabolic activity in the utilization of amino acids was observed for CO strains. The largest difference, up to 40%, was observed in the frequency of strains metabolizing L-histidine and glycyl-L-proline. Of the seven sugar acids tested, the three compounds used most frequently were D-galacturonic acid, D-gluconic acid, and D-glucuronic acid. These were metabolized by more than half of all strains. A small number of strains, specifically 5% and 23%, respectively, utilized glucuronamide and L-galactonic acid lactone as substrates. The study demonstrated a statistically significant (*p* < 0.05) higher activity of sugar acid metabolism by CO strains (Figure 3). Among EN bacteria, the frequency of strains metabolizing D-gluconic acid, L-galactonic acid lactone and D-saccharic acid was found to be twice as low as that of the CO ecotype (Figure 4A). No statistically significant differences were found in the metabolism of carboxylic acids (seven substrates) by the distinguished ecotypes of bacteria found in microbial mats. The three most frequently used compounds were propionic acid, acetic acid, and α-ketoglutaric acid, which were metabolized by 40% of all strains. Only approximately 10% of the strains utilized bromo-succinic acid as a substrate. Acetoacetic acid was sporadically metabolized exclusively by EN strains (5% of isolates). Formic acid was not a substrate for any of the bacteria studied. No significant differences were observed between CO and EN strains in the utilization of hydroxy acids. More than 50% of the strains used L-lactic acid, citric acid, L-malic acid, and β-hydroxy-D, L-butyric acid as substrates. Only 5% of the strains studied used α-hydroxybutyric acid and α-ketobutyric acid as nutrient sources. Over half of the cosmopolitan strains and nearly 40% of the endemic strains utilized dextrins, whereas none of the strains decomposed pectin's. Proteolytic properties (utilization of gelatin) were exhibited by nearly 60% of all isolates. Lipolytic properties (utilization of Tween 40) were observed in 18% of CO strains and 10% of EN strains.

### 3.3 Environmental tolerance

Functional profiling tests using the BIOLOG Gen III system revealed varying tolerance of the strains to environmental factors (Figure 4B). All isolates preferred a pH of 6, with 90% growing at 1% salinity. More than one-third of the strains grew in an acidic environment (pH 5). Nearly 36% of CO strains and 19% of EN strains exhibited halotolerant properties, and 5% of EN ecotype strains were also halophilic. Analysis of the impact of antibiotics on the bacterial strains showed that half of the isolates from microbial mats were resistant to three of the eight compounds used in the Gen III test. Four strains belonging to the CO ecotype: *Flavobacterium hydatis* (two strains), *Pseudomonas mandelii, Stenotrophomonas rhizophila* and two strains of *Janthinobacterium svalbardensis* and *Salinibacterium xinjiangense* from the EN ecotype were resistant to all the antibiotics used. Strains sensitive to all the antibiotics used, namely *Janthinobacterium lividum* and *Rhodoferax saidenbachensis*, were identified only in the CO ecotype. No significant differences were observed among the isolated ecotypes of the strains in their responses to the toxic compounds used in the Gen III test, although differences were observed in the potency of individual compounds. Most strains failed to develop in the presence of D-serine2, guanidine HCl, lithium chloride, sodium butyrate, and sodium bromate, with approximately 50% of the strains sensitive to potassium tellurite; 1% sodium lactate, Tetrazolium Blue, and Tetrazolium Violet exhibited weak toxic effects.

The metabolic activity of the strains under study, as measured by their ability to utilize organic substrates, differed between the two isolated ecotypes (Figure 2). CO strains showed significantly higher activity, with 50% metabolizing at least half of the substrates (36 positive responses). Three strains showed the highest metabolic activity: *Iodobacter limnosediminis, Pseudomonas mandelii*, and *Rhodoferax ferrireducens*, with 78%, 74%, and 71% positive responses, respectively. In the EN bacterial group, only a quarter of the strains were highly metabolically active, with three strains: *Arthrobacter psychrochitiniphilus, Salinibacterium xinjiangense* and *Flavobacterium antarcticum* exhibiting 64%, 63% and 61% positive responses, respectively. Both bacterial ecotypes included strains with low metabolic potential, utilizing up to 25% of the substrates. In the EN group, they accounted for one-third of all strains, with the lowest activity observed for *Iodobacter arcticus* (1% of substrates), *Janthinobacterium svalbardensis* (11% of substrates), *Flavobacterium xanthum*, and *Rugamonas violacea* (13% of substrates). In the CO group, two strains with such a metabolic profile were *Rhodococcus yunnanensis* (21% of substrates) and *Flavobacterium hydatis* (24% of substrates). Data analysis also revealed the presence of strains with broad metabolic profiles and high tolerance to environmental stresses. In the CO group, *Flavobacterium hydatis* was characterized by the assimilation of most substrates (69%), including the utilization of 71% of sugar compounds, and by growth in the presence of most toxic factors and antibiotics (87% positive responses). In the EN bacterial group, two such strains were identified: *Salinibacterium xinjiangense*, which could catabolize 63% of all metabolites and 65% of sugar compounds, and grow in the presence of all environmental factors, and *Exiguobacterium antarcticum*, characterized by 57% positive metabolic responses, including the decomposition of 50% of sugar compounds, and tolerance to 48% of environmental factors.

**Figure 5:**
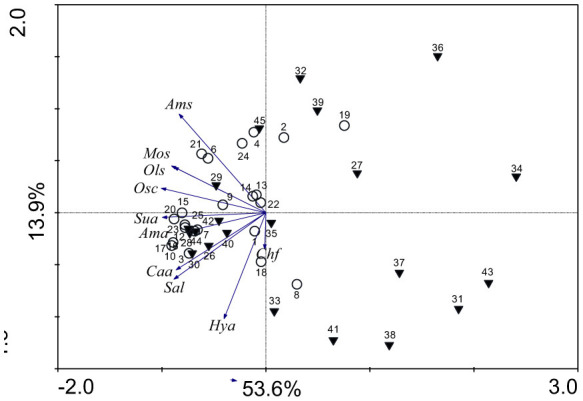
Considering that PCA plots have no physical units, the values on the axes are dimensionless and represent statistical distances that are the result of mathematical operations related mainly to the standard deviation, we propose leaving Figure 5 in its unchanged graphical form.

The analyses showed clear metabolic differentiation between strains classified into the distinct ecotypes CO and EN. The observed differences included the extent and intensity of available substrate utilization, suggesting specific metabolic preferences characteristic of each analyzed ecotype. Multivariate PCA showed that the studied metabolic activity explained 65% of the total variability within the analyzed strains (Figure 5). The first two principal components separated strains belonging to ecotypes CO and EN, indicating that metabolic differences are a significant factor differentiating these groups. This result highlights that metabolic variability is an important aspect characterizing the studied strains.

## 4 Discussion

Current research has shown that the taxonomic structure of polar mat microbial communities, shaped by distinct geographic and environmental conditions, results in diverse metabolic potential and the presence of specialized adaptations that enable survival and metabolic activity under extreme conditions. The group of strains studied, although representing only a limited fraction of the complex microbial community in mats, can be considered a model component of this community. Notably, the studied strains include organisms with diverse biogeographic ranges—from local to cosmopolitan forms—enabling examination of their functioning in a broader, global context. This approach captures both local adaptations and universal mechanisms of response to environmental stress.

The broad range of utilization of simple sugars, primarily glucose, mannose and fructose, by the polar mat bacteria studied may suggest the presence of efficient monosaccharide metabolism pathways. This reflects a specific adaptation to functioning within an active microbial loop, in which primary production determines the main source of organic carbon for microorganisms. As demonstrated, this mechanism is one of the form of adaptation to environments with limited carbon availability (Shen et al., 2017). In these ecosystems the microbial loop is a central element of the carbon and nitrogen cycles (Valdespino-Castillo et al., 2018). In microbial mats, sugars produced by cyanobacteria and other photoautotrophs constitute the primary fraction of dissolved organic matter (DOM). The presence of enzymes and catabolic pathways for carbohydrates, including monosaccharides, is a characteristic feature of many heterotrophic bacteria colonizing these environments (Bowman et al., 2016; Doytchinov and Dimov, 2022). The lack of clear differences in the ability to catabolize monosaccharides among isolated ecotypes of the strains may reflect the universal nature of these carbon sources and their fundamental importance in the metabolism of bacteria adapted to low temperatures. The high percentage of CO and EN strains utilizing D-maltose and D-trehalose as carbon sources highlights the crucial role of these oligosaccharides in the basic metabolism of heterotrophic bacteria in microbial mats. Although the transport of these compounds is energy-intensive, it is highly efficient, making them attractive substrates in nutrient-poor environments (Piegza et al., 2020). In microbial mats, these oligosaccharides, which are a component of DOM, are formed as a result of the degradation of extracellular polymeric substances (EPS), the breakdown of cyanobacterial and unicellular algal glycogen, and the hydrolysis of starch-like polysaccharides. These compounds are more stable and available than glucose at low temperatures, and their ecological importance indicates their role as key intermediate metabolites in the carbon cycle (Leyn et al., 2017). Trehalose deserves special attention, as it plays an important role in bacterial adaptation to polar conditions by stabilizing cell membranes, enzymes and structural proteins. Trehalose acting as an osmoprotectant, and serving as a reserve source of carbon and energy (Zhang et al., 2022). Microorganisms colonizing polar environments, including microbial mats, possess extensive gene sets that enable them to utilize compounds in the DOM pool, including sugar alcohols. This is confirmed by metagenomic and metatranscriptomic analyses of Antarctic microbial mats (Zaikova et al., 2019). These compounds play a particularly important role in polar ecosystems, where they can be synthesized and utilized by bacteria as part of their adaptation to osmotic stress and protection against freezing (Goszcz et al., 2025). The significant difference observed in the ability to utilize polyols (sorbitol, arabitol and glycerol) between the CO and EN strains studied may indicates the diverse metabolic potential of these ecotypes. The limited ability of EN bacteria to metabolize sugar alcohols may be related to adaptation to the trophic conditions of polar environments and possible genome optimization. The constant availability of simple compounds in DOM, which require low energy input for utilization, may support a more specialized metabolism in endemic polar strains. It has been suggested that these bacteria may exhibit partial genome simplification, potentially facilitating their functioning in a stable but trophically poor environment (Hwang et al., 2025). As a consequence of such a strategy is a potential reduction in genome size and a narrowing of the spectrum of carbon substrates utilized (Bowman et al., 2016; Chen et al., 2023). The higher activity of cosmopolitan bacteria in metabolizing amino sugars may suggest from greater metabolic plasticity in utilizing organic resources compared to the endemic bacterial community adapted to specific stress conditions. Survival strategies and stress-induced metabolic regulation may limit the rapid degradation of amino sugars by endemic microorganisms (Hu et al., 2018). Most likely, the low availability of N-acetyl-β-D-mannosamine (ManNAc), a component of eukaryotic glycoconjugates, in polar environments reduces the maintenance of efficient catabolic pathways for this amino sugar in the polar microorganisms studied. The energy-intensive transport and metabolism of phosphosugars at low temperatures may explain the poor utilization of these substrates by bacteria endemic to polar environments (Cao et al., 2020). The lack of metabolic activity in polar mat bacteria associated with pectin degradation, may be due to their primary origin from the cell walls of vascular plants, which are rare or seasonal in Antarctica (Smykla et al., 2007). Moreover, pectin metabolism requires a set of specialized extracellular enzymes, the synthesis of which is energy-intensive under polar conditions (Zhang et al., 2025). The current research confirmed reports by other authors (Cheng et al., 2022) that amino acids play a crucial role in the metabolism of Antarctic microorganisms, both as energy substrates and as components of protein biosynthesis or osmoregulation in response to stress conditions. Endemic bacteria may possess metabolic gene sets responsible for degrading of specific amino acids, while cosmopolitan taxa may retain more universal, complex metabolic pathways for a broader range of amino acids (Kleinteich et al., 2017; Du et al., 2025). A characteristic feature of polar bacterial communities is the low utilization of D-amino acids, whereas L-amino acids are more readily transported into the cell and are more efficiently metabolized by enzymes such as aminotransferases, deaminases, and dehydrogenases (Hill et al., 2011). The ability to utilize sugar acids, which enables bacteria to colonize niches poor in simple sugars, may indicates that cosmopolitan bacteria are effective at utilizing complex carbon sources. In polar microbial mats, sugar acids are key structural and metabolic components, occurring in high concentrations and serving several overlapping roles. In microbial mats, sugar acids (mainly uronic acids: glucuronic and galacturonic, but also, for example, mannuronic) are integral components of EPS produced by cyanobacteria and heterotrophic bacteria (Picazo et al., 2021). Sugar acids in microbial mats provide a structural scaffold, an environmental buffer, and an internal carbon store that enables complex consortia of microorganisms to function under extreme conditions. In addition, uronic acids stabilize mats during seasonal freezing and thawing conditions (Nagar et al., 2021). It has been demonstrated that D-galacturonic acid is a particularly important carbon source for bacteria in the studied microbial mats. (Picazo et al. 2021) found that this compound is present in the mucilage produced by cyanobacteria and other mat microorganisms, and that the bacterial communities analyzed were capable of degrading and metabolizing it. The low percentage of endemic strains capable of utilizing sugar acids observed in our study may reflect the limited availability of these substrates in the environment. Some polar bacteria prefer alternative carbon sources and may lack a complete set of sugar metabolism pathways, suggesting metabolic adaptation to local environmental conditions (Bowman, 2017). Cosmopolitan bacterial strains have access to sugar acids in many environments and retain genes and enzymes that metabolize uronates (Pick et al., 2015).

The similarity observed among the strains in their responses to environmental factors suggests high environmental adaptability. As shown by the present study, broad antibiotic resistance among strains derived from microbial mats is a common phenomenon (Górniak et al., 2025). An increasing number of reports indicate widespread antibiotic resistance among bacteria in various polar environments, including microbial mats (Králová et al., 2021; Marcoleta et al., 2022). Multidrug-resistant bacteria have been detected in polar terrestrial environments, glacial ice, and bottom sediments (Brown and Balkwill, 2009; Tam et al., 2015; Jara et al., 2020; Marcoleta et al., 2022). Studies show that the migration of cosmopolitan, often antibiotic-resistant, bacterial species into isolated polar ecosystems may promotes the transfer of resistance genes to the endemic microbial communities. The acquired nature of these characteristics is confirmed by the diverse resistance profiles of the strains studied, including those within the same species. This phenomenon appears common in microbial mats (Tallada et al., 2022). At the same time, it has been demonstrated that endemic species of polar environment bacteria are characterized by broad natural resistance to antibiotics (Marcoleta et al., 2022; Górniak et al., 2025).

The significant contribution of cosmopolitan bacterial species to the structure of polar microbial mats could be associated with their range expansion driven by climate change. The study showed the complex nature of the metabolic potential of the two distinguished ecotypes, both at the intraspecific and interspecific levels. (Du et al. 2025) note that an important aspect of these processes is the evolutionary optimization of genomes, which involves their gradual adaptation and improvement in response to environmental pressures.

## 5 Summary

In conclusion, this study is the first to examine the role of species' biogeographical ranges within polar microbial mats in the metabolic activity of these microbial communities. Our findings suggest that endemic bacteria may have metabolic properties for degrading a limited pool of essential substrates, potentially associated with a genome reduction strategy. In contrast, cosmopolitan taxa appear to maintain more universal and complex metabolic pathways. The higher activity of these bacteria, compared to endemic microbial communities, could be associated with their greater metabolic plasticity in transforming and utilizing organic resources. The observed differences in metabolic activity and resistance to environmental factors may partly reflect variation in the original biogeographic ranges of the studied species. Among the cosmopolitan species, strains characterized by a broad metabolic potential and high tolerance to environmental factors were identified. It can be assumed that cosmopolitan bacteria, due to their greater environmental tolerance and potentially faster growth, may influence the structure and functioning of local microbial communities. Their spread to isolated environments, such as polar regions, may promote the gradual homogenization of microbiomes on a global scale. This process may limit the participation of endemic species, potentially affecting nutrient cycling, nutrient regeneration, and bioconversion processes in polar ecosystems. Although our data do not allow conclusions regarding the genomic mechanisms underlying the observed metabolic differences, the current results suggest a possible relationship between the ecological strategy of the studied strains and the degree of genomic specialization. However, confirming whether any genome reduction is a direct result of adaptation to stable but trophically poor polar environments would require further analyses based on complete genomes and metagenomic, transcriptomic, and metabolomic data. Comparing strains with different biogeographic ranges would be particularly important, as it could determine the extent to which environmental pressures, geographic isolation, and substrate availability influence the metabolic potential of microorganisms. This issue represents an interesting avenue for future research on genome evolution, metabolic adaptation, and the functioning of microorganisms in extreme polar ecosystems. Current research contributes to the discussion on contemporary microbial biogeography, indicating that geographic ranges may be related to genomic adaptations and, consequently, to the metabolic and environmental adaptations of taxa.

## Data Availability

Datasets generated and analyzed during the current study are available from the corresponding author. Sequence data of studied bacteria strains have been deposited in the National Center for Biotechnology Information (NCBI) (https://pubmed.ncbi.nlm.nih.gov/) under accession numbers OR739452 - PQ517158.
